# Perceived Social Support and Mental Health Among Single vs. Partnered Polish Young Adults

**DOI:** 10.1007/s12144-014-9242-5

**Published:** 2014-06-29

**Authors:** Katarzyna Adamczyk, Chris Segrin

**Affiliations:** 1 Institute of Psychology, Adam Mickiewicz University, ul. A. Szamarzewskiego 89/AB, 60-568 Poznań, Poland; 2 Department of Communication, University of Arizona, Tucson, AZ 85721 USA

**Keywords:** Mental health, Mental health problems, Perceived social support, Single, Partnered, Young adults

## Abstract

The aim of this study was to examine whether young adults in nonmarital romantic relationships experience better mental health and lower levels of mental health problems compared to single young adults. In addition, the current study also tested the hypothesis that perceived social support mediates the association between relationship status (single vs. partnered) and mental health, and mental health problems. Five hundred fifty three participants (335 females and 218 males) aged 20–30 completed the Polish versions of General Health Questionnaire-28, Mental Health Continuum–Short Form, Social and Emotional Loneliness Scale for Adults–Short Form, and Multidimensional Scale of Perceived Social Support. Results indicated that single individuals reported lower emotional well-being than partnered individuals. No differences emerged between single and partnered individuals in regard to social and psychological well-being, as well in total well-being. Results also revealed no differences between single and partnered individuals in regard to somatic symptoms, anxiety and insomnia, social dysfunction, severe depression, and total mental health problems. Mediational analyses indicated the perceived social support mediates the association between partner status and mental health problems.

## Introduction

The engagement in a stable intimate relationship is one of the most prominent social roles in late adolescence and young adulthood (Roberts and Wood 2006), and constitutes one of the most prominent normative developmental tasks for young adults (Erikson 1980; Havighurst 1981). Romantic relationship involvement, per se, has generally been associated with greater well-being in adults (Umberson and Williams 1999). In general, there is considerable evidence that social ties and social support are positively and causally related to mental health, physical health, and longevity (Thoits 2011), and the lack of social attachments is associated with psychological and physical health problems, poor adjustment, and diminished well-being (Baumeister and Leary 1995). Furthermore, nonmarital romantic relationships are an important factor for emotional well-being in early adulthood (Simon and Barrett 2010). In turn, failure to establish and sustain a committed intimate relationship during young adulthood may have serious negative implications for well-being, both concurrently and later in the life span (Kiecolt-Glaser and Newton 2001).

Previous studies have shown marital status to be associated with better physical and psychological well-being. The typical focus of analysis in research on marriage and mental health has been internalizing problems (usually depression or symptoms of mental dysfunction), and externalizing problems such as substance use and abuse (especially alcohol) (Bierman et al. 2006; Uecker 2012). In prior research, single individuals were found to report higher levels of depression, anxiety, mood disorders, adjustment problems, and other forms of psychological distress, and a higher rate of alcohol-related problems (see Braithwaite et al. 2010; Johnston and Eklund 1984). In addition, popular social stereotypes that depict single individuals as miserable, lonely, unhappy, insecure, more neurotic, less satisfied with their lives, with lower self-esteem, less satisfied with their relationship status, and desiring to change their relationship status compared to individuals in relationships (DePaulo and Morris 2005; Greitemeyer 2009) enhance a negative view of single individuals’ mental health. That explanation for the mental health advantage of the married may differ depending on the outcome being examined, on the type of unmarried group under scrutiny (Bierman et al. 2006), or on relationship quality, age, attitudes toward civil status (DePaulo and Morris 2005). Therefore, the linkage between being in a committed relationship and mental health is not so strict and obvious, in particular when considering that with the increasing numbers of single people in the population of young adults in Poland and in other countries, singlehood is presently more normative and often is associated with positive outcomes such as happiness (Keith 2003), personal freedom, independence and self-decision making (Czernecka 2008), than a result of coercion or personal deficits.

Although the relationship between marital status and well-being has received substantial attention (Braithwaite et al. 2010), less attention has been given to the association between different relationship statuses and their outcomes during young adulthood (Soons and Liefbroer 2008). This neglected area calls for special attention because political, economic, demographic, and social-cultural changes in the last 20–30 years in Poland, Europe, and throughout the world have significantly changed the process of becoming an adult member of society (Brzezińska et al. 2011). Because of these changes, there is now a great diversity and individualization of life paths in young adulthood in the area of marital and family life. This diversity, in turn, is associated with alternative forms of marital and family life such as singlehood, cohabitation, single parent, and homosexual relationships (Slany 2006; Soons and Liefbroer 2008).

In Poland, as in many other countries, there is currently a clear tendency to postpone undertaking socially expected social roles typical for adulthood (Brzezińska et al. 2011). Recently in Poland there has been a decrease in marriages, an increase among young adults living in nonmartial relationships, a trend to postpone marriage until 30–34 years of age, and a noticeable increase of single individuals (from 27.10 % males in 2002 to 32.80 % in 2011, and from 19.10 % females in 2002 to 23.90 % females in 2011) (Demographical situation of Poland. Report of Government People Council 2012). These changes in the area of marital and family life forms in Poland as well as in other countries, contribute to the increasing significance of singlehood and nonmarital relationships in the lives of young adults. It is therefore valuable to compare single individuals with individuals in nonmarital romantic relationships as these relationships play a crucial role in young adults’ lives, their identity, self-concept, and psychological well-being (Simon and Barrett 2010).

In this article, we focused on never-married, heterosexual, mostly university young adults, who are entering into young adulthood, and we compared them with individuals in nonmarital romantic relationships, as suggested by Bierman et al. (2006). We also conceptualize well-being as not merely the absence of mental illness or psychological distress, but also the presence of positive feelings (emotional well-being) and positive functioning in individual life (psychological well-being) and community life (social well-being) (Lamers et al. 2011). Therefore, in the current study we investigated not only mental health in reference to mental illnesses such as depression or anxiety, but we also included the measurement of positive mental health as an important addition to the assessment of mental illness, and a better predictor of psychosocial functioning than a single diagnosis of mental illness (e.g., Lamers et al. 2011). Furthermore, because social resources (i.e., an individual’s actual social connections and his or her perception of the adequacy of these social connections in providing social support) play an important role in affecting mental health (Bierman et al. 2006), in the current study we also examined the role of perceived social support in mental health among single young adults and young adults in nonmarital romantic relationships. Presently, the vast majority of studies concerning marital status and its outcomes come from the USA. Consequently, there is a need for research from non-US samples in order to expand the scope and generalizability of the mostly American findings (Boski 2010). Therefore, the current study was conducted with a sample of Polish heterosexual young adults, many of whom were enrolled in a university, in order to expand the base of knowledge on the association between relational status and psychological well-being.

### Relationship Status and Mental Health

In general, prior research indicates that whether mental health is measured using diagnoses, symptoms, indicators of overall psychological well-being, or psychiatric treatment, currently married people enjoy the best health, followed by the never-married, and then the formerly married (Barrett 2000; Simon and Barrett 2010). Married men and women were found to have lower rates of mental illness than unmarried individuals (Gove 1972; Horn et al. 2013). As these findings illustrate, the study of well-being as a function of relationship status had traditionally compared unmarried people to their married counterparts. Simon and Barrett (2010) emphasized, however, that fewer studies have examined the association between nonmarital romantic relationships and mental health among adults, and those studies that exist compared adults in cohabiting relationships to those in married relationships rather than to those not currently in romantic relationships. Despite the deficit of research in this field, there is reason to believe that a variety of different relationship forms can be beneficial to well-being in adulthood (e.g., Braithwaite et al. 2010; Kamp Dush and Amato 2005; Soons and Liefbroer 2008). Several studies show that involvement in unmarried romantic relationships can be beneficial in terms of lower depression or other forms of psychological distress and higher life satisfaction (e.g., Ross 1995; Simon and Barrett 2010; Uecker 2012).

### Relationship Status, Mental Health and Perceived Social Support

One key element of social support is the perception of availability and quality of support from others (e.g., Procidano and Heller 1983). Perceived social support refers to perceptions of the extent to which people from one’s social network are available to provide social support (e.g., Demaray and Malecki 2002) and it may have a more significant effect than actual received social support (Pinquart and Sorensen 2003; Wethington and Kessler 1986). Social support is an important factor in mental health and well-being (e.g., Segrin and Domschke 2011; Zimet 1998). Social support may be directly related to health or it may have a mediating effect by enhancing health when an individual experiences high stress (Cotten 1999).

It is likely that there are direct and indirect associations between social resources, well-being, and relationship status (Barrett 1999). For instance, entering into a romantic relationship is likely to significantly enlarge an individual’s social network though immersion in the partner’s social network, thus increasing available support (Hurlbert and Acock 1990). Similarly, married and widowed individuals were also found to have networks of higher density, greater proportion of kin, and longer relationship duration than individuals who have never married or who have experienced marital disruption (Hurlbert and Acock 1990).

In contrast, the never-married are less likely to have a confidant and have lower levels of perceived support (Soons and Liefbroer 2008). In a Polish study conducted on a sample of young adults, single participants reported lower levels of significant others, family, and total perceived social support compared to counterparts in nonmarital romantic relationships. (Adamczyk 2013a). Consequently, it is reasonable to expect that one of the reasons for individuals in nonmarital romantic relationships. enjoying better well-being and single people experiencing higher distress is because of the greater social support available to those in partnered relationships.

### The Current Study

The present study is a part of a larger research project concerning subjective well-being and mental health of heterosexual single young adults and young adults in nonmarital romantic relationships in Poland (author Citation). This is to our knowledge the first study in Poland on relationship status, perceived social support, and mental health in young adults who are just entering into young adulthood. The aims of this study are twofold. First, we address the question of whether there are differences in mental health and mental health problems between young adults who are single and who are in nomarital romantic relationships. Based on previous research (e.g., Braithwaite et al. 2010; Kamp Dush and Amato 2005; Ross 1995; Simon and Barrett 2010; Soons and Liefbroer 2008), we expected that young adults in nonmarital romantic relationships will exhibit better mental health (i.e., higher emotional, social, and psychological well-being) and fewer mental health problems (i.e., somatic symptoms, anxiety, social dysfunction, severe depression) than single young adults. Our second aim is to contribute to the explanation of these differences in mental health. In particular, we focus on the role played by the perceived social support from family, friends, and significant others. We expected that perception of social support mediates the association between relationship status and mental health, and that it differs across single and partnered relationship statuses, thus partly explaining the differences in mental health for single and partnered relationship statuses.

## Methods

### Participants and Procedure

The study was carried out on a sample of 553 university students from different departments at Adam Mickiewicz University in Poznań, Poland and non-student individuals. One thousand questionnaires were originally distributed. A total of 688 participants returned questionnaires (response rate = a 68.80 %). Of these, 135 participants were removed because they were married, widowed, divorced, separated, older than 40 years old or because of incomplete data, yielding a final sample of 553 participants. The university students constituted 48.80 % of the total sample (*n* = 270), while non-student participants with a higher education level constituted 51.20 % of the total sample (*n* = 283). The age of participants ranged from 20 to 30 years old (*M* = 23.42, *SD* = 3.27). Women represented 60.60 % of the sample (*n* = 335) and men represented 39.40 % of the sample (*n* = 218). Participants resided in a large Polish city with a population exceeding 500,000. All the respondents were heterosexual, never married, and had no children, but declared that they wanted to have a lifetime partner in the future. Two hundred and seven participants (37.43 %) reported being single at the time of the assessment, while 346 participants (62.57 %) had a nonmarital romantic partner. Being single was defined as “not in a committed relationship for at least 6 or more months, but wanting to become committed in the near future (within the next year or so)”, and being in a nonmarital romantic relationship was defined as “in a nonmarital romantic relationship for at least 6 or more months, and wanting to be committed in the near future (within the next year or so)” (see Schachner et al. 2008).

The first author distributed the measures to students across different courses with the request to administer the questionnaires to their relatives, friends, and acquaintances.

The questionnaire packages were administered in classrooms to groups of 20 to 30 students at a time and participation was voluntary. An explanation of the study’s purpose was given along with assurance to students that all information provided would remain anonymous and confidential. Students who volunteered to participate received extra credit toward their final course grade.

#### Materials

The questionnaire presented to the study participants was comprised of the following instruments:

##### Demographic Questionnaire

These questions were designed to obtain general descriptive information about participants’ background such as their age, gender, major, education, and current relationship status.

##### General Health Questionnaire-28

(GHQ-28; Goldberg and Hillier 1979) (Polish adaptation–Goldberg et al. 2001). The General Health Questionnaire measures symptoms of non-psychotic psychiatric disorders (Goldberg and Hillier 1979). The GHQ-28 scale was derived from the original 60-item version of the questionnaire mainly for research purposes but it is also often used as a measure of psychological well-being (e.g., Goldberg and Williams 1988). GHQ-28 consists of four 7-item scales: somatic symptoms, anxiety and insomnia, social dysfunction, and severe depression. Respondent are asked to compare their recent psychological state with their usual state on a 4-point (1 = *not at all*, 2 = *no more than usual*, 3 = *rather more than usual*, 4 = *much more than usual*). In the current study the bimodal scoring procedure (0, 0, 1, 1) was applied. Using the conventional bimodal GHQ scoring method, there is a range of 0–28 with a score above a threshold of 4 indicative of psychiatric disorder. In the present study the internal consistency for the subscales was *α* = .75, *α* = .80, *α* = .77, *α* = .79, and *α* = .89 for Somatic symptoms, Anxiety, Social dysfunction, Severe depression, and for the Total scale, respectively.

##### Mental Health Continuum–Short Form

(MHC–SF; Keyes 2009) (Polish adaptation–Adamczyk 2014). It is the short form of the Mental Health Continuum (MHC-SF) derived from the long form (MHC-LF), which consisted of 40 items measuring emotional well-being, 18 items that measured the six dimensions of Ryff’s model of psychological well-being, and 15 items total that measure the five dimensions of Keyes’ model of social well-being. The MHC-SF consists of 14 items that were chosen as the most prototypical items representing the construct definition for each facet of well-being. Three items were chosen (happy, interested in life, and satisfied) to represent emotional well-being, six items (one item from each of the six dimensions) were chosen to represent psychological well-being, and five items (one item from each of the five dimensions) were chosen to represent social well-being. Respondents are asked to indicate how they have been feeling during the past month using the scale ranging from 0 (*never*) to 5 (*every day*). The MHC-SF also provides a categorical diagnosis of mental health, namely a diagnosis of *flourishing* is made if someone feels 1 of the 3 hedonic well-being symptoms “every day” or “almost every day” and feels 6 of the 11 positive functioning symptoms “every day” or “almost every day” in the past month. *Languishing* is the diagnosis when someone feels 1 of the 3 hedonic well-being symptoms “never” or “once or twice” and feels 6 of the 11 positive functioning symptoms “never” or “once or twice” in the past month. Individuals who are neither “languishing” nor “flourishing” are then coded as “*moderately mentally healthy*.” The short form of the MHC has shown excellent internal consistency (>.80) and discriminant validity in adolescents and adults in the U.S., in the Netherlands, and in South Africa. In the present study the internal consistency for the subscales was high: *α* = .87 for Emotional well-being, *α* = .80 for Social well-being, *α* = .81 for Psychological well-being, and *α* = .89 for Total mental health.

##### Multidimensional Scale of Perceived Social Support

(MSPSS; Zimet et al. 1988) (Polish adaptation–Adamczyk 2013b). The MSPSS measures the adequacy of one’s perceived social support from three domains: family, friends, and a significant other. There are four items per subscale, each with response options ranging from 1 (*very strongly disagree*) to 7 (*very strongly agree*). Higher scores on each of the subscales indicate higher levels of perceived support, and a sum of the three scales yields a global satisfaction with perceived support score. Zimet et al. (1988) investigated and found internal reliability estimates of .88 for total score, and .93, .92, .93 for Friends, Family and Significant Other subscales, respectively. In the present study internal consistency for the subscales was very high: *α* = .94, *α* = .91, *α* = .95, and *α* = .92 for the Significant Other, Family, Friends, and Total support scales, respectively.

## Results

We first examined the hypothesis that individuals in committed relationships would experience better mental health (i.e., higher emotional, social, and psychological well-being) than single young adults. In order to examine possible mean differences between single individuals and individuals in nonmarital romantic relationships in regard to mental health, a one-way multivariate analysis of variance was used. The analysis resulted in a significant multivariate effect of relationship status on mental health, Wilks’s Λ = .96, *F* (3,549) = 7.98, *p* < .001, η^2^ = .04.

Follow-up univariate analyses presented in Table 1 indicated that individuals in nonmarital romantic relationships scored higher on emotional well-being than single individuals. At the same time, no differences emerged between the both groups in regard to social and psychological, or well as to total well-being. With a sample of this size, the smallest difference that could be detected with power = .80 is *d* = .24 which is close to what Cohen (1988) characterized as a “small” effect.

**Table 1:** Means and standard deviations on mental health and mental health problems by relationship status

	Single sample (*N* = 207)	Partnered sample (*N* = 346)	*F value*	*η2*
Variables	Mean (SD)	Mean (SD)		
Multivariate test			7.98***	.04
Mental health				
Emotional well-being	9.46 (3.38)	10.45 (3.06)	12.37***	.02
Social well-being	10.57 (5.22)	9.90 (5.61)	1.92	.00
Psychological well-being	16.82 (6.46)	17.45 (5.57)	1.47	.00
Total well-being	36.86 (13.13)	37.80 (12.05)	.75	.00
Multivariate test			1.42	.01
Mental health problems				
Somatic symptoms	1.85 (1.88)	2.07 (1.93)	1.83	.00
Anxiety and insomnia	1.80 (2.00)	1.84 (2.06)	.06	.00
Social dysfunction	1.15 (1.62)	1.09 (1.67)	.22	.00
Severe depression	.76 (1.46)	.59 (1.28)	2.00	.00
Total mental health problems	5.57 (5.36)	5.60 (5.50)	.01	.00

*** *p* < .001

In addition, we examined possible differences between single individuals and individuals in nonmarital romantic relationships on the categorical diagnosis of mental health described by Keyes (2009). Using the scoring method described by Keyes (2009) participants’ scores on the MHC-SF were converted into these three categories. These were then included in a 2 (partnered/single) × 3 (languishing, moderate, flourishing) multiple samples chi-square test. Results of this test indicated significant differences in frequencies of relationship status by mental health functionality, *χ*2 = 8.22, df = 2, *p* < .05, Cramer’s *V* = .12.

The frequencies in Table 2 indicate that 12 % of the single participants were in the languishing group versus only 6 % of those in the partnered group. For the moderate group, 65 % of the single participants were in this category as were 75 % of the participants in nonmarital romantic relationships. Finally, 22 % of the single participants were in the flourishing group and 19 % of the participants in nonmarital romantic relationships were in this group. Overall, it appears that a greater proportion of the single people than partnered people are in the languishing group, and more people in nonmarital romantic relationships than single people are in the moderate mental health functionality group.

**Table 2:** The distribution of mental health categories by relationship status

	Mental health categories					
	Languishing		Flourishing		Moderately mentally healthy	
Relationship status	*n*	%	*n*	%	*n*	%
Single status	25	12 %	45	22 %	137	65 %
Partnered status	20	6 %	66	19 %	260	75 %
Total sample	45	8 %	111	20 %	397	72 %

*N* = 553. Single status (*n* = 207); Partnered status (*n* = 346)

Percentage are provided for a given category of relationship status

Next, we tested for differences in mental health problems (i.e., somatic symptoms, anxiety, social dysfunction, severe depression). A one-way multivariate analysis of variance resulted in a non-significant multivariate effect of relationship on mental health problems, Wilks’s Λ = .99, *F* (4,548) = 1.42, *ns*, *η* ^*2*^ = .01 (see Table 1). Single individuals and individuals in nonmarital romantic relationships did not differ on the mental health problems, i.e., somatic symptoms, anxiety and insomnia, social dysfunction, severe depression, and total mental health problems.

**Fig. 1:**
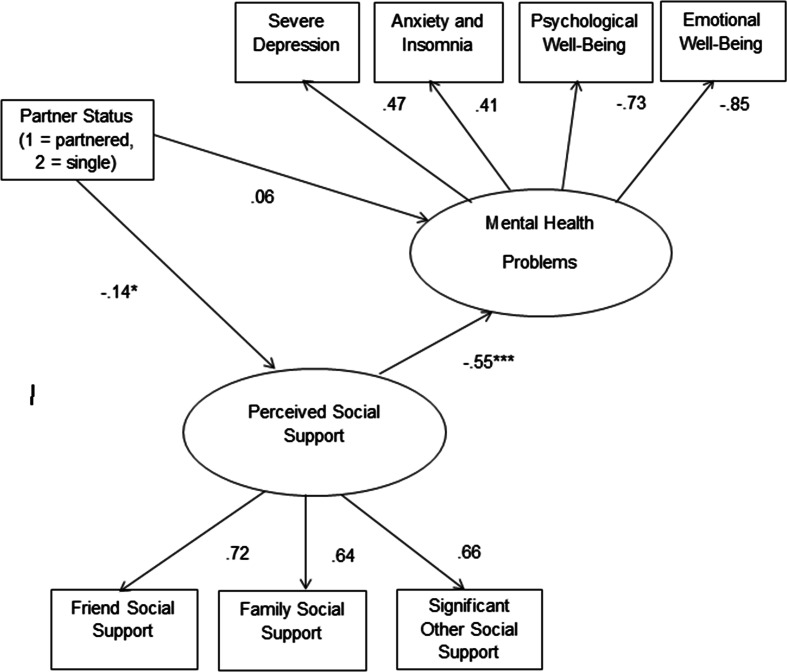
Structure model of partner status, perceived social support, and mental health. Note. Figure values are standardized regression coefficients. For ease of presentation, error terms have been omitted from the model. *p < .05. **p < .001

Structural equation modeling in AMOS 20.0 was used to test whether perceived social support mediated the association between relationship status and mental health (hypothesis three). For this analysis, partner status (dummy coded as 1 = being in a nonmarital romantic relationship, 2 = single) was the exogenous variable, perceived social support score was treated as the mediating variable, and mental health was the dependent variable. Perceived social support was treated as a latent variable indicated by amount of support available from friends, family, and a significant other. Similarly, mental health was represented as a latent variable, indicated by anxiety and depression from the GHQ and by psychological well-being and emotional well-being from the MHC-SF scale. Prior to evaluating the fit of the structural model, a measurement model was analyzed in which correlations were specified between all possible pairs of observed or latent variables in the model. The initial results indicated that the fit of the model could be improved by specifying an additional correlation between the anxiety and depression error terms of the mental health latent variable, and between the partner status observed variable and the error terms associated with friend and significant other social support. With these correlations specified, the model provided a good fit to the sample data, *χ*2 = 44.14, df = 15, *p* < .001, *χ*2/df ratio = 2.43, NFI = .96, CFI = .98, RMSEA = .06, 90 % c.i. = .04–.08. Next we analyzed the structural model depicted in Fig. 1.

Because we were interested in comparing the direct effect of partner status on mental health problems to the corresponding indirect effect, through perceived social support, we specified both the direct and indirect effects in the model. The significance of the indirect effect was estimated with a bias-corrected bootstrapping procedure based on 2000 bootstrap samples to estimate standard errors and confidence intervals around the indirect effect.

The results of this analysis suggested that this model provided a good fit to the sample data, *χ*2 = 43.11, df = 15, *p* < .001, *χ*2/df ratio = 2.87, NFI = .96, CFI = .98, RMSEA = .06, 90 % c.i. = .04–.08. In this model, the path from partner status to perceived social support was significant (*β* = −.14, *p* < .001) indicating that people in nonmarital romantic relationships

reported greater overall social support than people who did not have a partner. Similarly, there was a significant association between perceived social support and the mental health latent variable, *β* = −.55, *p* < .001, reflecting the fact that perceived social support was negatively associated with anxiety and depression and positively associated with psychological and emotional well-being. As predicted, there was a statistically significant indirect effect of partner status on mental health problems, through perceived social support, *β* = .08, *p* < .05. In contrast, the direct effect of partner status on mental health problems, controlling for perceived social support, was not statistically significant, *β* = .06, ns. This indicates the perceived social support mediates the association between partner status and mental health problems.

To determine whether the indirect effect of partner status on mental health, through social support, was influenced by inclusion of the significant other social support scale, we re-estimated this indirect effect, after deleting that scale from the social support latent variable. The indirect effect without the significant other social support factor (*B* = .091, *p* = .037) was virtually unchanged from the indirect effect in the original model (*B* = .098, *p* = .028). Therefore, the indirect effect of partner status on mental health, through social support, is not simply a consequent of lower significant other support among those without a romantic partner.

## Discussion

The main purpose of this study was to investigate differences in mental health across single and partnered statuses, and also to examine whether perceived social support mediates the association between relationship status and mental health. Because most prior research focused on marital status and on adults of all ages (Soons and Liefbroer 2008), we compared heterosexual young adults, many of whom were enrolled in a university, who were single to those in nonmarital romantic relationships. Following suggestions to assess multiple aspects of mental health and illness (e.g., Bierman et al. 2006), we measured both positive (e.g., emotional well-being, psychological well-being) and negative (e.g., depression and anxiety) aspects of mental health.

It was predicted that young adults in nonmarital romantic relationships would exhibit better well-being than single young adults. This hypothesis was only partially supported.

The results indicated that individuals in nonmarital romantic relationships reported higher emotional well-being, but there were no differences in regard to social and psychological well-being. The prediction that individuals in nonmarital romantic relationships would be underrepresented in the languishing category of mental health and overrepresented in the flourishing category, relative to single individuals, was partly borne out by these results.

The proportion of single people in the languishing group was two times as much as the proportion of people in nonmarital romantic relationships in the languishing group. However, proportionally, the two groups were equally represented in the flourishing group. Thus, it can be concluded that the major distinction between the single and partnered groups is the greater propensity of single individuals to be languishing in their mental health.

Second, we expected that young adults in nonmarital romantic relationships would exhibit fewer mental health problems (i.e., somatic symptoms, anxiety, social dysfunction, severe depression) than single young adults. This hypothesis was not supported as our analyses revealed no differences between single individuals and individuals in nonmarital romantic relationships in regard to mental health problems. This pattern of results contradicts prior findings indicating that in general commitment in romantic relationships is associated with benefits for people’s mental health and sense of well-being (Kamp Dush and Amato 2005). These results may reflect the fact that singlehood is more normative among young adults and perhaps not as deleterious to their mental health as has been the case historically.

Although there was greater emotional well-being among participants in nonmarital romantic relationships and a somewhat higher proportion of single individuals than individuals in nonmarital romantic relationships in the “languishing” mental health group, most of the tests comparing single people and people in nonmarital romantic relationships in mental health yielded no significant differences. In Polish society, as in many societies around the world, singlehood is becoming more common among young adults because young adults are waiting longer to marry, and fewer people are getting married, compared to past times (e.g., Czernecka 2013; Jones et al. 2012; Such-Pyrgiel 2014). One obvious effect of this demographic change is that singlehood is a less stigmatized relational state for young adults in the modern age. Furthermore, the reasons for singlehood in many people may be more voluntary than nonvoluntary (Prabhakar 2011). It therefore may be understandable why the lack of a romantic partner may not be associated with extensive psychological problems for participants in this study as singlehood is presently assumed to be an expression of individualization and individualistic attitudes, and the expanded freedom of people’s choice (Poortman and Liefbroer 2010).

The results obtained in the present study support observations made by some researchers that negative associations with singlehood (e.g., popular social stereotypes describing single individuals as miserable, lonely, unhappy, insecure, more neurotic, less satisfied with their lives compared to individuals in relationships (DePaulo and Morris 2005; Greitemeyer 2009), may not be accurate. Rather, contemporary singlehood may represent choice, and may be associated with positive outcomes, for instance, for happiness (Keith 2003). Also, there is evidence that as long as people marry by their desired age, mental health remains well (Carlson 2012). Carlson’s research showed that it is only those who are unmarried at a point in the lifespan where they wanted to already be married (plus those who married earlier than desired) who are at risk for psychological morbidity. Additionally, for some people remaining single is merely a time limited phase in life before getting married or being committed in a serious relationship (Kaiser and Kashy 2005). For instance in a study on single Mormons, individuals perceived their singlehood as “a temporary stage on the road towards marriage” (Darrington et al. 2005, p. 656). Similarly, in a Polish study by Palus (2010), most of single young adults indicated that they perceive their singlehood as a temporary state eventually leading to marriage. This perception of singlehood as temporary, in turn, may be related to the young age and so called life potential in anticipation of future times (Worach-Kardas 1990). Because the typical participant in current study was only 23 years of age, it would be reasonable to assume that most had not sought to be in a long-term committed relationship yet, and therefore they perceive their single status as a temporary state. If so, this could partly explain the general lack of significant differences when comparing single individuals and individuals in nonmarital romantic relationships.

Relationship status was predicted to have an indirect effect of psychological well-being through greater social support to those in partnered relationships. This hypothesis was supported, despite the lack of a direct effect of relationship status on psychological well-being. There was, however, a significant association between being in a nonmarital romantic relationship and reporting greater perceived social support, and between perception of social support and greater psychological well-being/fewer symptoms of mental health problems. Collectively, these two associations are the components of the indirect effect of relationship status on mental health. These results are consistent with others in the literature showing that social support is a key component in promoting better mental health and avoiding psychological distress (e.g., Bierman et al. 2006; Segrin and Domschke 2011; Zimet 1998), and that social support may be an important factor for explaining why single individuals and individuals in nonmarital romantic relationships exhibit differential risk for psychological morbidity (Bierman et al. 2006). Quite simply, this may be because individuals in nonmarital romantic relationships feel that they have greater social support available to them than single individuals do (Adamczyk 2013a; Turner and Marino 1994).

It should also be noted that among the three types of perceived social support (family, friends, significant other) the most prominent difference between the single and partnered participants was on the significant other dimension, understandably. Means for single and partnered participants were 16.84 and 20.60, respectively, *t*(551) = 10.64, *p* < .001. However, when removing this variable from the structural model depicted in Fig. 1, the indirect effect from relationship status to mental health was still statistically significant. This indicates that there is something more to the deficiencies in social support experienced by single people that explains their decreased psychological well-being. The fact that single people also reported less family social support (*M* = 16.94) than partnered individuals did (*M* = 17.72), *t*(551) = 2.00, *p* < .05, suggests that their social support deficits are somewhat pervasive and not constrained exclusively to the domain of significant other relationships.

The present results should be considered in light of several limitations. First, the cross-sectional data do not allow for inferences about the direction of associations between relationship status and mental health. Although prior research provides support for the linkage between relationship status and mental health, the *social selection* hypothesis indicates that better-adjusted, healthier individuals become and remain married (Horn et al. 2013). Therefore, the associations and models tested in the present study need to be replicated in a longitudinal design. Second, the sample was made up primarily of university students and other well-educated individuals. The lack of lower income, less educated, and older adults in the sample limits the generalizability of these findings. It would be useful for future research to replicate these findings in a more demographically diverse sample. Third, the sample used in the current study consisted only of heterosexual, never-married and childless young adults. Singles are a diverse group (Cotten 1999) and these results may not generalize to widowed, divorced, or separates adults for example. Fourth, it would be useful for future researchers to measure quality, rather than mere involvement in, nonmarital relationships as analogous research on married people has shown marital quality to have salutary effects on health and well-being (DePaulo and Morris 2005). Finally, because about half of the sample included university students and half were individuals recruited by university students, there may be some nonindependence in the observations. Although university students were specifically instructed to not recruit their romantic partners into the study, they were allowed to recruit friends or other young adult family members. It is possible that this decreased the observed variance in the measures included in this investigation.

This results of this study question whether single young adults are at risk for greater mental health problems than counterparts in nonmarital romantic relationships. At this point in the lifespan, being single may not have the stigma that it once did in the past, and it is possible that many young adults remain single voluntarily while they pursue educational and career goals. This may explain why the mental health problems and psychological well-being were generally comparable between single individuals and individuals in nonmarital romantic relationships. At the same time, these results clearly illustrate that even though relationship status does not have a direct effect on mental health, it does have an indirect effect. Individuals in nonmarital romantic relationships enjoy greater social support, and that social support, in turn, is associated with greater psychological well-being and fewer symptoms of mental health problems.
